# Flow Rate-Independent Multiscale Liquid Biopsy for Precision Oncology

**DOI:** 10.1021/acssensors.2c02577

**Published:** 2023-02-20

**Authors:** Jie Wang, Robert Dallmann, Renquan Lu, Jing Yan, Jérôme Charmet

**Affiliations:** † Institute for Advanced Materials, School of Material Science and Engineering, Jiangsu University, Zhenjiang 212013, China; ‡ Division of Biomedical Sciences, Warwick Medical School, University of Warwick, Coventry CV4 7AL, U. K.; § Department of Clinical Laboratory, Fudan University Shanghai Cancer Center, Shanghai 200032, China; ∥ Holosensor Medical Technology Ltd., Suzhou 215000, China; ⊥ WMG University of Warwick, Coventry CV4 7AL, U.K.; # School of Engineering − HE-Arc Ingénierie, HES-SO University of Applied Sciences Western Switzerland, 2000 Neuchâtel, Switzerland

**Keywords:** liquid biopsy, microfluidics, circulating tumor cells, mass transport, precision oncology, multiscale

## Abstract

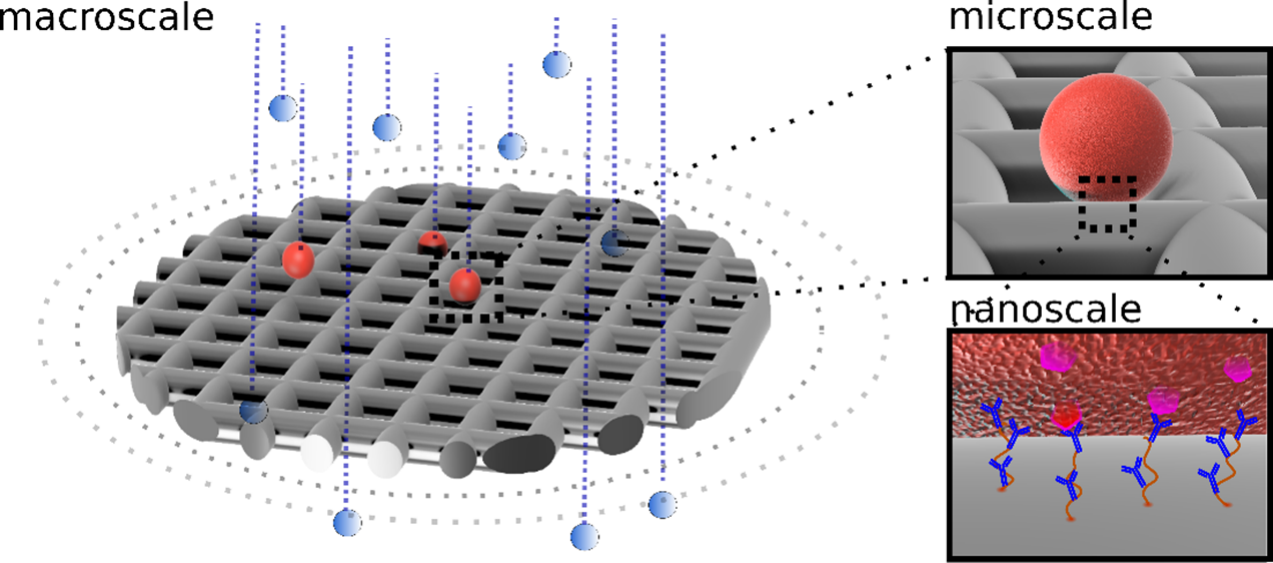

Immunoaffinity-based liquid biopsies of circulating tumor cells (CTCs) hold great promise for cancer management but typically suffer from low throughput, relative complexity, and postprocessing limitations. Here, we address these issues simultaneously by decoupling and independently optimizing the nano-, micro-, and macro-scales of an enrichment device that is simple to fabricate and operate. Unlike other affinity-based devices, our scalable mesh approach enables optimum capture conditions at any flow rate, as demonstrated with constant capture efficiencies, above 75% between 50 and 200 μL min^–1^. The device achieved 96% sensitivity and 100% specificity when used to detect CTCs in the blood of 79 cancer patients and 20 healthy controls. We demonstrate its postprocessing capacity with the identification of potential responders to immune checkpoint inhibition (ICI) therapy and the detection of HER2 positive breast cancer. The results compare well with other assays, including clinical standards. This suggests that our approach, which overcomes major limitations associated with affinity-based liquid biopsies, could help improve cancer management.

Liquid biopsies have the potential to transform cancer management through noninvasive, real-time feedback on patient conditions.^1−4^ Circulating tumor cells (CTCs) that are released from primary and/or distant tumors into the bloodstream^5^ are seen as a particularly useful source of information to improve clinical outcomes (patient prognosis, real-time responses to therapeutic interventions, and monitoring of tumor recurrence), guide drug discovery, and advance our understanding of cancer progression, metastatic cascade, and minimal residual diseases.^6−9^ However, the capture of such cells from blood is technically challenging due to their low abundance, typically 1–10 CTCs per mL.^10−12^ This constraint imposes the processing of a large sample volume, usually between 4 and 10 mL, to retrieve enough cells. Consequently, the ideal device must combine high capture efficiency and high throughput.

Among the many CTC enrichment strategies, those relying on size differences to discriminate cancer cells from healthy blood cells have recently gained in popularity.^3^ This is because these approaches are capable of very high throughput using devices that are relatively simple to fabricate and operate. However, it has become apparent that the CTCs captured by such devices may fail to recapitulate their native biological complexity and heterogeneity. For example, such devices may miss small-sized CTCs^13^ that are correlated with aggressive metastatic progression in patients.^14^ Even though several multistep solutions have been developed recently to address these issues,^15−18^ they are more complex than their counterparts based on physical capture alone. Therefore, it would be ideal to develop an affinity-based solution that harbors the same simplicity and throughput as their cells size-based counterparts.

In affinity-based devices, the capture of cells typically relies on the interaction between the cell’s surface markers and complementary antibodies tethered to the channel walls. The current issue with such devices is the reliance on channels or structures with dimensions on the order of the target cells size. Even though small channels dimensions enhance the interaction between the target cells and the capture elements,^2,3,10,19−21^ they also limit the flow rate and hence the throughput. Indeed, most affinity-based microfluidic devices proposed to date have an upper limit of a few mL h^–1^ above which the capture efficiency drops significantly (typically up to 2 mL h^–1^ as reported in a number of reviews^3,11,22,23^). In addition to the flow rate limitation, affinity-based liquid biopsy devices are usually complex due to their inherent small sizes and channel geometries (e.g., refs (23−25)). Finally, such devices do not always allow for easy postprocessing since the cells are typically surface-bound inside the chip and not readily accessible or retrievable.

Our study, which revisits widely accepted yet misleading common knowledge in microfluidics, allowed us to propose a novel strategy that addresses these issues. We conceptualized a simple yet widely applicable solution that relies on a scalable macroscale mesh with nanofunctionalized micropores (Figure 1a–d). Our solution addresses the (1) flow rate dependence, (2) complexity, and (3) postprocessing limitations simultaneously. First, we demonstrate an optimized capture efficiency above 75%, independent of the flow rate, up to 200 μL min^–1^ (or 12 mL h^–1^), which is approximately an order of magnitude higher than most commonly reported values for surface-based capture in conventional microfluidic devices.^11,22,24−26^ Second, the production of our device does not rely on complex microfabrication processes, and its operation only requires an external pumping system to process manually loaded buffers and samples (Figure 1e–i). Third, it allows for easy, off-chip functionalization before assembly and, importantly, allows for simple postprocessing of the captured CTCs. Figure S1 shows the detailed process steps.

**Figure 1:**
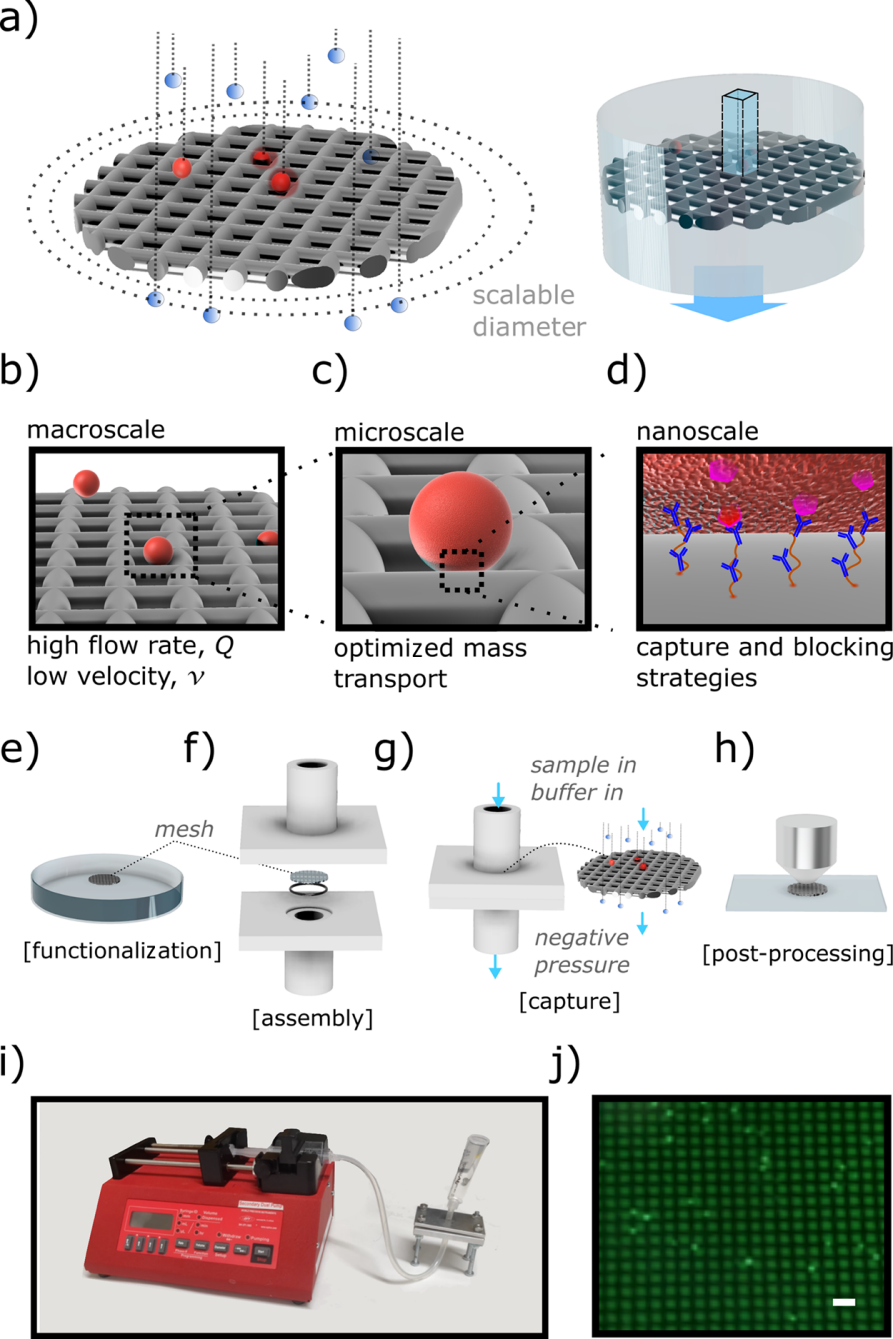
Architecture of the device. The core of the device is a scalable micromesh—not to scale (a) that enables the decoupling and independent optimization of biosensing-relevant length scales. The resulting device offers a macroscale channel to maintain low fluid velocity while running samples at high flow rate (b), a microscale mesh whose dimension enhances interactions with the target cells (c), and a nanofunctionalized coating that enables high capture efficiency and low nonspecific interaction (d). The main operations, including functionalization (e), assembly (f), capture (g), and postprocessing (h), are made easier by the fact that the mesh can be easily removed from its holder, allowing access for pre (e) and postprocessing (h) as shown in (j) that displays GFP expressing MCF-7 cells observed after capture, scale bar 40 μm. The assay can be performed using widely available laboratory equipment (i).

We validate the device clinically by isolating CTCs in 4 mL blood samples from 79 cancer patients. The sensitivity and specificity of our device using conventional staining protocols to identify the CTCs were of 96 and 100%, respectively, against a group of healthy controls (*n* = 20). We also demonstrate its postprocessing capacity with the identification of potential responders to immune checkpoint inhibition (ICI) therapy and the detection of HER2 positive breast cancer. The results compare well with other assays, including clinical standards, and show the potential applicability of our simple multiscale, flow rate-independent liquid biopsy strategy for cancer management.

## Results

### Decoupling Length Scales for Flow Rate-Independent Capture and Device Optimization

To better understand the flow rate limitation that seems to affect affinity-based solutions, we concentrate on the Peclet number (*Pe*), a dimensionless metric used to optimize biosensors.^27−29^ The Peclet number measures the ratio of the convection rate over the diffusion rate. Applied to the context of rare events (such as CTCs in blood), a small Peclet number (≪1) is ideal as it promotes interaction between the target cell and the functionalized surface. In other words, it is a condition that maximizes cell capture. In most microfluidics publications, the Peclet number is written as *Pe* = *Q*/*DL* (e.g., refs (27, 28, 30)), *Q* being the flow rate, *D* the diffusion coefficient, and *L* the characteristic length scale. This notation emphasizes the flow rate limitation. Indeed, the equation suggests that flow rate cannot be increased without decreasing the Peclet number (and hence the capture efficiency). However, the Peclet number can also be written as *Pe* = *vL*/*D*, which implies a fluid velocity (*v*) limitation. This notation allows us to conceptualize a situation with a low Peclet number and high flow rate, provided the latter can be decoupled from the fluid velocity.

The fact that the Peclet’s number flow rate limiting notation is so widely accepted is because flow rate and velocity are coupled via the channel’s cross-section in conventional channels (see *v* = *Q*/*A* and Supp. Mat. for details). However, it suffices to introduce structures that have two inherent length scales to decouple the two parameters. Here we use meshes with microscale pore size and macroscale diameter mounted in a channel of matching size. In this configuration, the velocity can be kept low (to maintain *Pe* ≫ 1 and hence optimal capture) for any flow rate. Indeed, for a given flow rate, the fluid velocity can be reduced by increasing the cross-section of the mesh *A*_s_ as shown in *v* = *Q*/*A*_s_. We achieve this by cutting the commercially available mesh to the right diameter (see the Materials and Methods section for details). This approach does not compromise the micron-size length scale (pore size) necessary for optimal mass transfer or the nanoscale critical for functionalization strategies.

**Figure 2:**
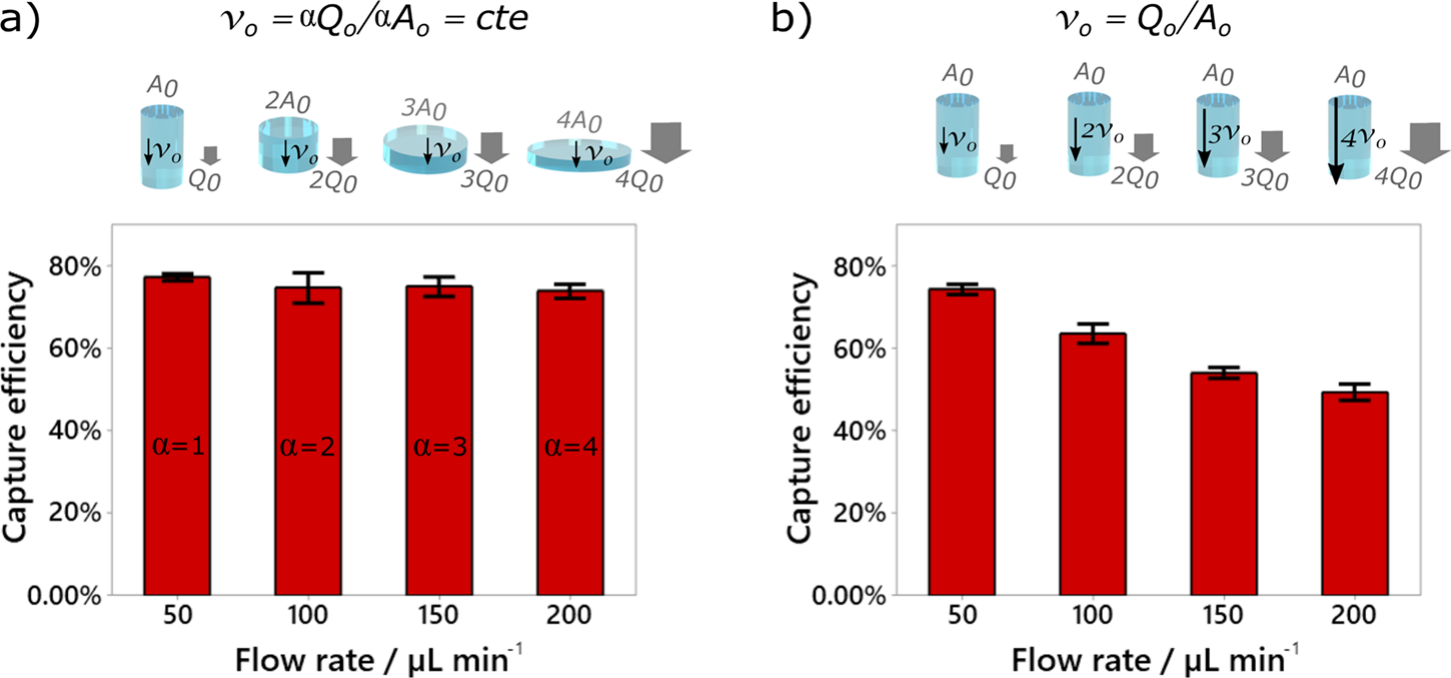
Flow rate independence. Panel (a) shows that capture efficiency can be kept constant (as confirmed by one-way ANOVA [*F* = 0.37, *p* = 0.775] and post-hoc Tukey’s test (Figure S2)) as long as the velocity is kept constant. This is achieved by scaling the surface area and the flow rate by the same factor, as given by *v* = α*Q*/α*A*. In comparison, one observes a significant capture efficiency decrease (one-way ANOVA [*F* = 38.04, *p* < 0.0001]) when the diameter of the mesh is kept constant (b). The measurements were performed using MCF-7 cell lines spiked in buffer solutions. The bars represent the standard error based on three individual measurements.

**Figure 3:**
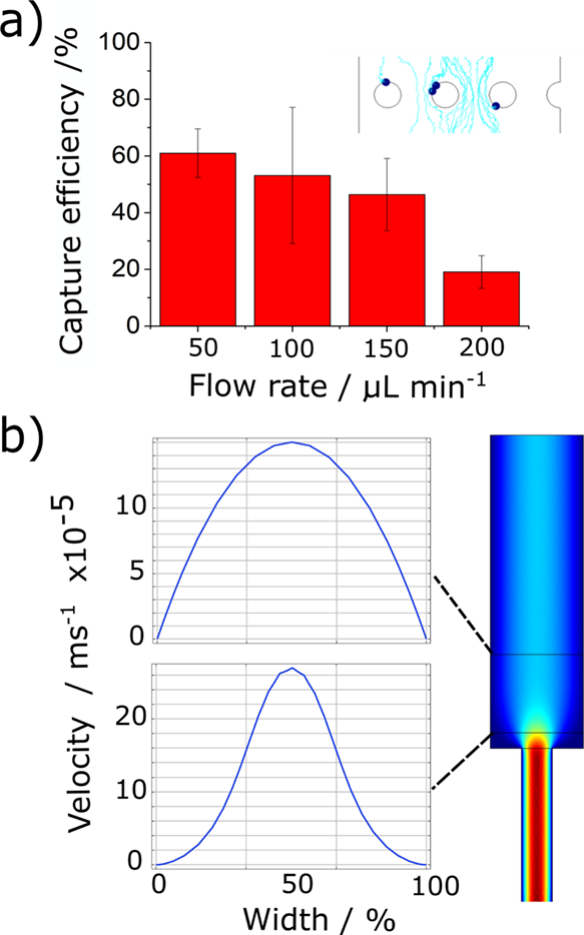
Comparative (semiquantitative) multiphysics simulations. (a) shows the effect of the flow rate on the capture of particles on the mesh (cross-section). The capture efficiency decreases progressively as the flow rate increases. The values observed are based on at least three individual measurements in the same conditions. (b) shows the effect of restrictions in the vicinity of the mesh (cross-section). If the mesh is too close to a diameter restriction, the velocity increases locally, thereby negatively impacting the capture efficiency.

Taking the concept even further, we show how to achieve a flow rate-independent capture. Indeed, an arbitrary velocity *v*_a_ can be kept constant provided the flow rate and the diameter are scaled by the same factor α as shown in *v*_a_ = α*Q*_a_/α*A*. This is demonstrated experimentally by measuring the device’s capture efficiency at different flow rates (Figure 2). The capture efficiency is given by the ratio of the captured to the introduced target cells. We used GFP expressing MCF-7 breast cancer cell line (as shown after capture in Figure 1j) and the functionalization procedure described below on a 15 × 20 μm pore size mesh. First, we defined the diameter-dependent optimal flow rate *Q*_o_. It is the flow rate yielding the maximum capture efficiency before drop-off using a mesh with a fixed diameter. A constant capture efficiency of 75% is observed until *Q*_o_ = 50 μL min^–1^ before decreasing significantly as shown in Figure 2b (and Figure S3 in Supplementary Materials), for a mesh of 6 mm diameter. This behavior is consistent with other affinity-based liquid biopsies^11,22,24^ and with our simulations (Figure 3). The optimal velocity of our system, *v*_o_ = *Q*_o_/*A*_s_ (with *Q*_o_ = 50 μL min^–1^ and *A*_s_ ≅88.3 mm^2^ ( ⌀ = 6 mm)), is thus *v*_o_ = 2.95 × 10^–5^ m s^–1^. This velocity can be kept constant for any flow rate provided *Q*_o_ and *A*_s_ are multiplied by the same factor, α. Figure 2a shows no significant difference in capture efficiency for α = 1–4, i.e., for flow rates ranging from 50 to 200 μL min^–1^, as determined by one-way ANOVA [*F* = 0.37, *p* = 0.05]. A post-hoc Tukey’s test shows that there is no statistical difference between any of the flow rates (Figure S2). This result is in stark contrast with Figure 2b that shows a strong dependency on the flow rate, as determined by one-way ANOVA [*F* = 38.04, *p* < 0.0001], when the diameter of the mesh is kept constant. Graphs with flow rates down to 20 μL min^–1^ (Figure S3) and details of the post-hoc Tukey’s tests (Figures S2 and S4) are provided in Supplementary Materials. More practically, this approach can be used to find an optimum capture efficiency given a target flow rate, *Q*_t_. Indeed, a simple rule of three suffices to define the optimal mesh cross-section, *A*_st_, as defined by *A*_st_ = *Q*_t_/*v*_0_ = *A*_s_*Q*_t_/*Q*_0_ (the initial surface area times the ratio of the target to the initial flow rate).

Comparative (semiquantitative) multiphysics simulations (COMSOL Multiphysics 5.5) were performed to optimize the device (Figure 3). The effect of the flow rate on the capture efficiency is shown in Figure 3a, which represent the cross-section of a mesh in a channel. The particle tracing module was used to evaluate the flow rate dependence of the capture efficiency for discrete events to represent individual cells (Figure 3). Similar results were obtained from simulations using diluted species (Figure S5). In either case, the results provided qualitative data whose trend aligned well with experiments. We also used the simulation to optimize the position of the mesh in the device. Figure 3b highlights the effect of channel diameter restrictions, which can locally increase the velocity across a mesh if it is positioned in its proximity. Since an increased velocity reduces the capture efficiency, it is important to position the mesh sufficiently far away from any diameter restrictions.

### Nanobranched Polymers for Capture Optimization

We used thiol-terminated nanobranched polymers tethered to a gold-coated micromesh and functionalized with antibodies against specific cell surface receptors to confer high capture efficiency and high specificity to our device. First, we targeted epithelial cell adhesion molecules (EpCAM) that are characteristically overexpressed in a range of epithelial cancers but not in blood cells.^31^ EpCAM has already been used in a number of affinity-based liquid biopsy studies^11,22,31−33^ and therefore provides a good standard for comparison. Nanobranched polymers can accommodate multiple antiEpCAM antibodies alongside blocking molecules, increasing the probability of antigen-to-antibody contact and minimizing nonspecific interaction.^34−36^

**Figure 4:**
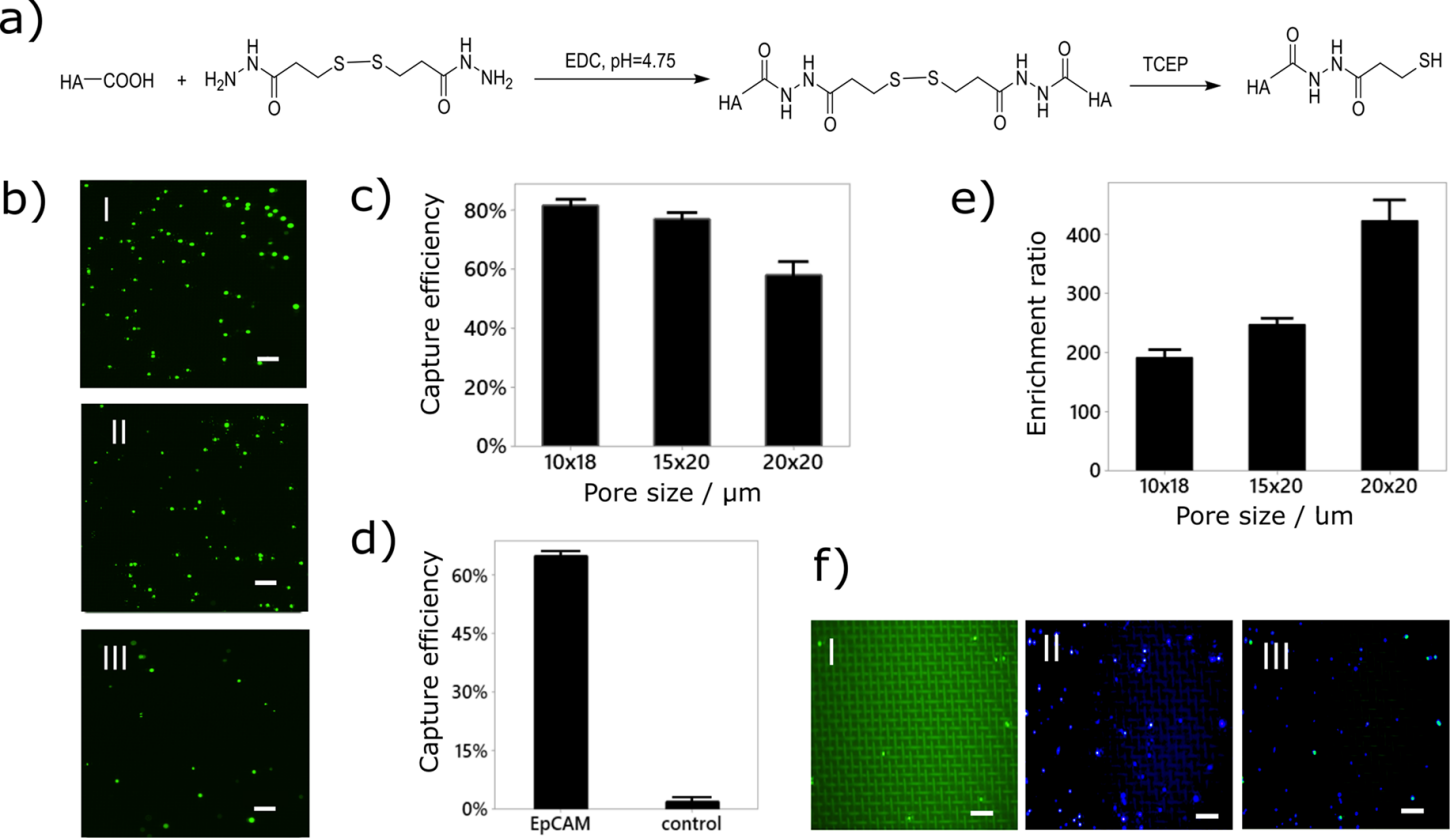
Nanofunctionalization strategies and performance of the device. (a) Nanobranched sulfhydryl hyaluronic acid (HA-SH) synthesis steps. (b) Micrographs of captured MCF-7 cells on the mesh after perfusion with different concentrations (approximately 150, 100, and 50 cells per mL for I, II, and III, respectively). (c) Effect of different meshes pore sizes (10 × 18, 15 × 20, and 20 × 20 μm) were evaluated on cell capture efficiency. (d) Control experiment using antiIgG to replace antiEpCAM antibodies shows that far fewer CFSE-stained MCF-7 cells are captured, confirming the good immunocapture specificity (e). Adding Jurkat cells as background, we evaluated the enrichment ratio as function of the pore size. (f) Typical example of the captured cells. (I) CFSE-stained MCF-7 cells, (II) DAPI+ cells comprised mostly of background Jurkat cells, and (III) merged image. Scale bars represent 200 μm. The error bars represent the standard error based on triplicate measurements.

The simple design of the device enables seamless removal and mounting of the microscale mesh (Figure 1e–h), such that its functionalization can take place in optimal conditions, with minimum waste of precious material. Figure 4a represents the simplified polymer synthesis steps. Detailed descriptions of the polymer synthesis and functionalization steps are given in the Materials and Methods section. Briefly, gold-coated micromeshes were incubated in sulfhydryl hyaluronic acid (HA-SH) for 2 h, followed by activation using EDC/NHS in MES buffer (pH = 6). After 30 min, antiEpCAM antibodies were incubated at 37 °C for 2 h to finalize the functionalization of the mesh (Figure 4a). Antibody dosage and incubation times were systematically examined to optimize binding efficiency (Figure S6 in Supplementary Materials). After the reaction, blocking molecules were added (1 h incubation time) to reduce nonspecific binding (details provided below).

We first optimized our device using buffer solutions spiked with a known number of EpCAM expressing MCF-7 cells. The capture efficiency of the device is given by the ratio of captured cells to the total number of cells added to the solution. Before capturing, MCF-7 cells were loaded with intracellular live cell dye (CFSE) to simplify their counting and differentiation from background. To optimize the cell culture parameters, we compared the effect of different harvest reagents and the influence of passage number on the EpCAM receptor’s integrity by performing flow cytometry and immunostaining (Figure S7). Having optimized these parameters, we evaluated the capture efficiency.

A functionalized 6 mm diameter mesh was mounted into the holder with a fluidic channel of matching (φ = 6 mm) dimension. A syringe pump was then connected to the holder via medical grade tubing. After priming the device with buffer to the top of the mesh, medium spiked with appropriate cells was added to the open reservoir. The solution was then withdrawn through the mesh at a flow rate of 50 μL min^–1^, which corresponds to the optimal flow rate for a 6 mm diameter mesh as explained above. Next, fresh medium was added to the reservoir to wash off nonspecifically bound cells and ensure that only the cells captured by affinity binding stay on the mesh. Finally, the mesh was removed and observed under a microscope to count the captured cells. Figure 4b shows cells captured on the mesh with varying initial cell concentrations. The simplified process steps are shown in Figure 1e–h (a more detailed version is presented in Figure S1).

Capture efficiency of 58% (σ^2^ = 6.3%) is obtained with a 20 × 20 μm pore size micromesh (Figure 4c). In contrast, antibodies tethered directly to the gold-coated mesh via Traut reagent result in capture efficiencies below 20% (Figure S8). These result are in line with previously reported studies that noted improved capture efficiency when using nanobranched polymers.^36^Figure 4c also shows that the capture efficiency increased with decreasing pore sizes (at 0.05 level, the population means are significantly different, using one-way ANOVA, *F* = 15.12), even though the 10 × 18 and 15 × 20 μm pore size meshes cannot be considered statistically different using post-hoc Tukey’s test (Figure S9). This increased efficiency is attributed to the higher probability of cells interaction with the functionalized surface. Micromeshes with 10 × 18 μm pore sizes result in capture efficiency up to 81.6% (σ^2^ = 1.4%) and correspond to typical values reported for a range of affinity-based microfluidic approaches.^37,38^ Even though such pore sizes can also filter larger CTC clusters, they are generally too large for capturing single CTCs.^39,40^ This further confirms that the captures observed in our case are due to affinity binding. It is interesting to note that the capture efficiency does not scale linearly with the projected surface area of the mesh, i.e., the active functionalized surface as “seen” by the cells. The reduction observed for smaller mesh pore size is attributed to higher local velocity due to the decrease in total open area compared to the optimized flow rate for larger pore size. Indeed, the total mesh open area is reduced by 19% with the 10 × 18 μm pore sizes compared to the 20 × 20 μm pore size, resulting in a velocity increase of 24% for the same flow rate. This suggests that the flow rate *v*_o_ should be optimized for each pore size.

To further evaluate the specificity of our device, we compared the capture efficiency of MCF-7 cells with MDA-MB-231, T24, and NCl-H1975 control cells (Figure S10a). The relative capture efficiencies are in agreement with the EpCAM expression level of each cell type (Figure S10b). Figure 4d shows that far fewer MCF-7 cells are captured using antiIgG as control on a 20 × 20 μm pore size micromesh, confirming the good immunocapture specificity of our approach.

Next, we quantified the enrichment of target cells, defined as the ratio of target to background cells detected (on the mesh) divided by the ratio of target to background cells in the sample.^10^ For this purpose, we repeated the capture efficiency experiment with the addition of ∼1 × 10^6^ Jurkat cells (EpCAM-), corresponding to a ratio of about 1:10^4^ MCF-7: Jurkat cells. Using BSA (1%) as blocking solution, enrichment ratios corresponding to 192, 248, and 424 were observed for 10 × 18, 15 × 20, and 20 × 20 μm pore size meshes, respectively (Figure 4e,f). Even though similar values have been reported elsewhere, higher enrichment ratios are also possible, highlighting a potential for improvement of our device.^38,41^ The population means are significantly different, using one-way ANOVA (*F* = 28.04, *p* = 0.001), confirming a reduction in nonspecific interaction with decreasing total functionalized area as expected. However, a post-hoc Tukey’s test reveals that the 10 × 18 and 15 × 20 μm pore size mesh cannot be considered statistically different. This can be attributed to a combination of the increase in velocity reported above for the 10 × 18 μm pore size mesh that reduces interaction probability and the associated increased shear stress that promotes removal of nonspecifically bound cells.^11,42^ Using trimethoxylsilane (50%) blocking solution instead of BSA did not result in any significant improvement in enrichment (Figure S11a) or changes in capture efficiency (Figure S11b) for meshes with 15 × 20 μm pore size. It is also noted that no significant differences in capture efficiency were observed between the measurement with and without background cells in the same conditions (*t*(4) = 1.0409; *p* = 0.3567).

### Validation Using Clinical Samples

Having characterized the performance of our novel device with immortalized cell lines, we validated its utility using clinical samples. In brief, we evaluated its performance in the first step of our study, then assessed its postprocessing capabilities using a subset of patients (step 2), and finally compared it with clinical standards in the third step.

First, we recruited 79 cancer patients and 20 healthy controls from Fudan University Shanghai Cancer Center and Changzheng Hospital (ethical approval #050432-4-1911D). Demographic details of the study population are given in Table S1. To evaluate the applicability of our device and to reflect the diversity of clinical cases that could benefit from liquid biopsy, we have selected patients with 10 different cancers, including nonsmall-cell lung cancer and breast cancer. The volume of blood sampled was 4 mL and processed according to the local clinical standard prior to detection (see the Materials and Methods section for details). Meshes with 20 × 15 um pore size, 6 mm diameter, and HA-SH polymer with BSA blocking were used with the optimized flow rate of 50 μL min^–1^ (as described above), resulting in a total sample processing time of less than 10 min (including priming and washing steps).

**Figure 5:**
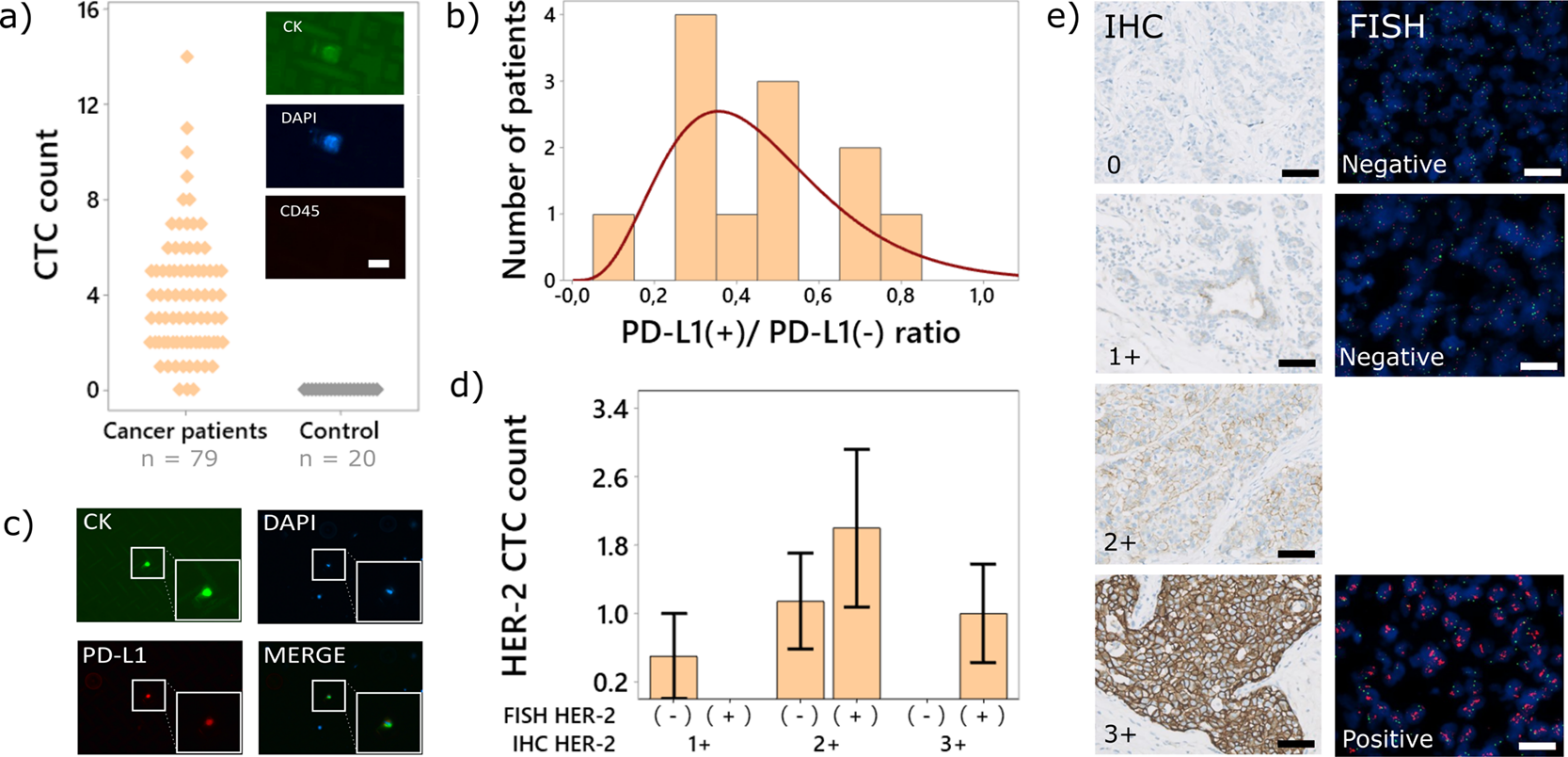
Clinical validation of the device on two cohorts in three separate studies. The first study, shown in panel (a), with 79 cancer patients with a range of different cancer types and 20 healthy controls was used to characterize the performance of our device. CK+, DAPI+, and CK- cells were identified as CTCs (inset), scale bar 20 μm. A subset of patients (*n* = 33) from the initial cohort were selected to evaluate PD-L1 expression to identify potential responders to ICI therapy. The distribution of PD-L1+ CTCs to CTCs is shown in (b). PD-L1 expression was evaluated using standard secondary immunostaining (c), scale bar 100 μm. To evaluate our device against clinical standards, we selected 26 breast cancer patients and tested them for HER2 positivity using secondary immunostaining postprocessing. We compared our results (d) with the histological scoring of IHC and FISH (based on Table S2), two standard clinical assays. Representative images are shown in (e). Scale bar 40 μm.

The cells captured on the mesh (by the antiEpCAM antibody), staining for CK^+^/CD45^–^/DAPI^+^, were identified as CTCs. Among the cancer patients, 96% (76/79) had at least one CTC and about 4% (3/79) had more than 10 CTCs. Using the same enumeration criteria, we tested the healthy controls and detected no CTCs (20/20). The results are summarized in Figure 5a. To define the sensitivity and specificity of our device, we performed a logistics regression based on the scikit-learn Python package. Using 30% of the data as training set and 70% for classification, we calculated the sensitivity (96%) and specificity (100%) of our device. As confirmation, we generated the receiver operation characteristic (ROC) that yielded an area under the curve (AUC) value of 0.979 (not shown). These excellent values are due to the dual selection (immuno-capture and staining) inherent to our assay and are in line with results reported for devices using similar approaches.^11^

In the past decade, studies on CTCs have gone beyond simple enumeration. The analysis of feature-rich CTCs, that may possess attributes of the primary tumor as well as metastasis, can provide clinically actionable information.^1−4^ For example, the expression of PD-L1 in tumor tissues, used as a biomarker for the selection of patients eligible for ICI therapy, is also evaluated using CTCs.^43−45^ The upregulation of PD-L1 enables cancer cells to evade immune response by inhibiting the activation of immune cells. ICI therapies target antiPD-L1/PD1 proteins to block the inhibition of immune cells, thereby reactivating the immune system. To further demonstrate the applicability of our device in this clinical context and, in particular, to evaluate its postprocessing capabilities, we selected 33 patients from the original cohort (*n* = 79) for the evaluation of PD-L1 expression using immunofluorescence labeling (Figure 5b,c). This step was performed after isolation of CTCs using antiEpCAM antibodies as described earlier. On this basis, 36% of patients (12/33) had PD-L1 expressing cells. Among them, the largest proportion of patients (*n* = 4) only had 30% of PD-L1+ CTCs as shown in Figure 5b. Based on the data from the NSCLC patients (*n* = 21), we detected a median of four CTCs per 4 mL (range: two to nine CTCs per 4 mL), among which 48% (*n* = 10) harbored at least one PD-L1+ CTC. The total number of CTCs in 4 mL compares well with at least two commercial systems^46^ and confirms the efficacy of our approach.

For a final validation study, we used our device to identify HER2 positive breast cancer patients based on the detection of HER2 positive CTCs. We compared our results with immunohistochemistry (IHC) and fluorescence in situ hybridization (FISH), two standard methods approved by the U.S. Food and Drug Administration (FDA). HER2 protein overexpression and/or HER2 gene amplification is found in about 20% of breast cancers and is associated with tumorigenesis, increased risk of metastasis, and poor prognosis.^47,48^ Importantly, these markers can be used to identify patients that could benefit from targeted therapies, such as trastuzumab, pertuzumab, and T-DM1. In this study, we selected 26 breast cancer patients from the initial cohort (*n* = 79). They were tested for HER2 status using clinical standard procedures (IHC and FISH) performed on tissue (solid biopsy) and compared with HER2 positive CTCs captured using our device (liquid biopsy). IHC is typically used as a screening test with IHC 0 and IHC 1+ considered as negative, IHC 2+ equivocal and IHC 3+ as positive (Figure 5d). FISH is considered more reliable, but it is more complicated and expensive, and is therefore normally used to determine the status of IHC 2+ equivocal cases.^47−49^

The CTCs were captured using the protocol described for the PD-L1 study, with fluorescently labeled antiErbB2/HER2 antibodies. The results are summarized in Figure 5c. According to the above criteria, 10 patients were identified as HER2 positive using standard approaches (IHC and/or FISH) against 14 identified based on the HER2 positive CTCs. The data shows a consistency (positive rate) of 54.17% (13/24) with the IHC and 80% (8/10, *p* = 0.0156) with FISH (Table 1). It is a promising result especially since FISH is considered a superior assay. We also note that our device captured HER2+ CTCs when the FISH or even IHC assay produced nonequivocal negative results (7 and 1, respectively). Similar findings were reported elsewhere^50^ and may be due to intratumoral HER2 heterogeneity.^49^ More studies, beyond the scope of this manuscript, will be necessary to evaluate the clinical relevance of HER2+ CTCs.

**Table 1:** Comparison between the Clinical Standards (IHC and FISH) and Our Device

subject	patients	patients with HER-2 positive CTC	positive rate (%)	chi-square *p*
HER2 IHC				0.8921
1+	2	1	50.0	
2+	21	11	52.4	
3+	3	2	66.7	
HER2 FISH				0.0156
(−)	16	5	31.3	
(+)	10	8	80	

## Discussions

We have characterized and validated the utility of a new multiscale enrichment device that enables flow rate-independent capture and processing of CTCs. We have achieved this by introducing a mesh structure that has two inherent length scales and, thus, eliminates flow rate as the main limiting factor in affinity-based liquid biopsy. The resulting device offers (1) a macroscale channel to run samples at high flow rate while maintaining low fluid velocity, (2) a microscale mesh that promotes interaction with the target cells, and (3) a nanofunctionalized surface that enables high capture efficiency and low nonspecific interaction. Using micromeshes with 15 × 20 μm pore size and HA-SH nanobranched polymer, we have reached >76% capture efficiency, which is comparable to a number of microfluidics-based liquid biopsies.^37,38^ However, unlike conventional microfluidics approaches, we have also demonstrated how our device can be scaled to allow for optimal capture efficiency for any flow rate, demonstrated here up to 12 mL h^–1^ (200 μL min^–1^).

Importantly, our device is easy to fabricate and assemble, its operation does not require specialist equipment, and its architecture allows for simple pre and postprocessing. Hence, our approach can be seamlessly integrated into conventional laboratory workflows, including in demanding clinical environments. To demonstrate this, we validated our device using clinical samples. Using data from 79 cancer patients and 20 healthy donors as control, our device yielded 96% sensitivity and 100% specificity, which is comparable to other approaches relying on a combination of capture and staining.^11,51^ Then we used it to identify potential responders to PD-L1 ICI therapies. In comparison to commercially available liquid biopsy approaches, in the context of nonsmall-cell lung carcinoma, our device performed favorably.^46^ Finally, we validated our approach against clinical standards in the context of HER2 positive breast cancer on a cohort of 26 breast cancer patients. In particular, we observed an 80% correspondence with FISH positive results.

Finally, we also note that our functionalization strategy is compatible with the addition of further antibodies or capture molecules for improved cell isolation efficiency^52^ but also for other liquid biopsies. In addition, our nanobranched polymer is amenable to modification and may allow for the integration of cell release strategies (e.g., refs (26, 35)), which will enable further downstream analysis, including next generation sequencing. In conclusion, our multiscale, flow rate-independent multiscale liquid biopsy approach has the potential to help drive significant advances in diagnosis, prognosis, and fundamental studies for a range of conditions.

## Materials and Methods

### Device Fabrication and Preparation

#### Fabrication

Micromeshes of different pore sizes (10 × 18, 15 × 20, and 20 × 20 μm) were obtained from Zhongxin Hairu Ltd., China (Cat. nos.: 1000 635, 800 635, and 635 635, respectively). They were cleaned in 70% ethanol using ultrasound for 5 min, rinsed in deionized water, and then dried using nitrogen (N_2_). Meshes were gold-coated (50 nm both sides) using magnetron sputtering and cut to size (e.g., 8.8 mm diameter mesh for the 6 mm diameter mesh holder) using clean surgical forceps. The mesh holders were fabricated using 3D printing or conventional machining (Figures 1 and S12).

#### Preparation and Cleaning of the Mesh

MilliQ water, 25% ammonium hydroxide, and 30% hydrogen peroxide were mixed in a clean beaker (5:1:1 ratio) and heated to 75 °C. The cut meshes were submersed in this solution for 5 min and afterward washed in MilliQ water and 99% ethanol before drying with N_2_ and then transferred into a clean Petri dish for functionalization.

### Nanofunctionalization

#### HA-SH Nanobranched Polymer Synthesis

40 mL of MES solution (Aladdin, Cat. no.: M108952, pH = 4.75, 0.1 M) was slowly added into the single-mouth flask. 200 mg of sodium hyaluronate (Bloomage BioTechnology, Cat. no.: HA-TLM, molecular weight: 3.9 W) was then added into the flask and stirred (magnetic stirrer 400 rpm) until the sodium hyaluronate was fully dissolved (5–8 min). Then, 60 mg of DTP (Frontier scientific, Cat. no.: D13817) was added into the flask and stirred thoroughly until completely dissolved. 120 mg of EDC (Sinoreagent, Cat. no.: 30083834) powder was added into the solution which was then stirred at 400 rpm at room temperature for 5 h. Finally, 150 mg of TCEP (Sigma, Cat. no.: C4706) was added into solution. After overnight (about 16 h) stirring, the HA-SH was filtered (0.22 μm filter) and collected.

#### Mesh Functionalization

Clean meshes (up to 18) were submerged in 3 mL of HA-SH in a 5 mL centrifuge tube and orbital shook at 200 rpm for 2 h to form thiol–Au bonds between HA-SH and the mesh. After washing three time with 3 mL of PBS, the meshes were submerged in 3 mL of SH-PEG-COOH (Toyongbio, Cat. no.: P003002) for 1 h to react the unbonded Au. Then the meshes were washed three times with 3 mL of PBS, dried, and put in a 24-well plate (one mesh per well). Activating reagents (55 μL per mesh in MES (pH = 6, 0.05 M)), comprised of 1-(3-dimethyl aminopropyl)-3-ethyl carbodiimide (EDC, Sigma, Cat. no.: 03449): 0.609 mg/mesh (35 μL) and n-hydroxysuccinimide (NHS, Sigma, Cat. no.: 56485): 0.348 mg/mesh (20 μL), were added to the surface of each HA-SH functionalized gold mesh and incubated at room temperature for 30 min. After incubation, the gold meshes were removed and washed three times with 500 μL of PBS. The meshes were then dried and moved to new 24-well plate. The capture solution was prepared by adding 7 μL of antiEpCAM antibody (1:2000, #324202, Biolegend, CA, USA) to 50 μL of MES solution (pH = 6, 0.05 M) and subsequent vortexing. The capture solution (57 μL) was then added onto a mesh and placed at 37 °C and 5% CO_2_ in an incubator for 2 h to allow for an amide bond to be created between the antiEpCAM antibody and HA-SH. Then, the mesh was removed and washed twice with 1 mL of PBS. A blocking solution to minimize nonspecific interaction (450 μL of 1% BSA solution (w/v%)—Sigma (B2064-50G)) was added to the gold mesh and returned to the incubator for 1 h. After washing with PBS (Hyclone), meshes were submerged in 500 μL of cryoprotectant (45% sucrose (w/v%, Sinoreagent, Cat. no.: 10021463) and 15% glycine (w/v%, Sinoreagent, Cat. no.: 62011516) in Tris–HCL (Sangon Biotech, Cat. no.: B548127-0500, 1 M, pH = 8.0)), precooled to −20 °C for at least 1 h until it was solidified, and then lyophilized. The lyophilized mesh was then sealed with desiccant and stored at −20 °C ready for use.

### Cell Culture and Labeling

#### Cell Culture

Human breast cancer (MCF-7, MDA-MB-231), urinary bladder cancer (T24), lung cancer (NCl-H1975), and monocytic (Jurkat) cells were obtained from iCell (China). All cells were cultured as recommended using phenol-red free Dulbecco’s Modified Eagle’s Medium (DMEM) (Gibco, NY, USA) supplemented with 1% l-glutamine (Life Technologies, CA, USA), 10% fetal bovine serum (FBS, Gibco), and 1% penicillin/streptomycin (Corning, VA, USA), with the exception of MCF-7, which were grown in 50:50 phenol-red free DMEM:F12 (Gibco) supplemented with 1X B27 (Gibco), 5 mg/L insulin (MBL International Corp., MA, USA), 20 μg/L basic fibroblast growth factor (bFGF, Shenandoah Inc., PA, USA), 20 μg/L epidermal growth factor (EGF, Shenandoah Inc., PA, USA), 1% penicillin/streptomycin (Corning, VA, USA), 0.5 mg/L hydrocortisone (Sigma Aldrich, MO, USA), and 2.5 mM l-glutamine (Life Technologies, USA).

#### Cell Labeling

For staining, cells were trypsinized and washed twice with PBS before being stained with cell tracker (CellTrace CFSE Cell Proliferation Kit) following the manufacturer instructions and resuspending cells in 1 mL culture media. Green fluorescent protein (GFP) expressing MCF-7 cells were generated by lentiviral transduction with pWPI as previously described.^53^

#### EpCAM Expression

Flow cytometry was performed using BD Accuri C6 flow cytometer (BD Biosciences, USA). EpCAM mouse antihuman FITC conjugated antibody was used for epithelial marker expression (Cat. # 347197, BD Bioscience, USA).

#### Spiking Assay

MCF-7 cells below 80% confluence were trypsinized (Life Technologies, USA) and washed two to three times in PBS. Then, cells were accurately counted and viability was determined with a cell counter (Countess, ThermoFisher, USA) in cell culture media with serum; the indicated numbers of cells were resuspended in 4 mL buffer for analysis together with ∼1 × 10^6^ background cell lines.

### Material from Clinical Studies

Cancer patients and control groups were recruited at Fudan University Shanghai Cancer Center and Changzheng Hospital, China (ethical approval #050432-4-1911D) after providing informed consent. Patients in this cohort may have received preoperative surgery or systematic anticancer treatment but must have been enrolled in this cohort at least 30 days in advance.

For processing in our novel devices, at least 4 mL of blood from cancer patients and healthy individuals were collected and stored in EDTA tubes and the blood was tested within 6 h. Before detection of CTC, blood was processed according to the local clinical standard. Briefly, the blood was diluted 1:1 in PBS (pH = 7.0) and then carefully transferred to a sterile 15 mL centrifuge tube which contained prewarmed density gradient separation solution (4 mL, Dakewe Biotech, Shenzhen, China). This layered liquid tube was centrifuged to 700 g at room temperature for 20 min. The PBMC layer was pipetted into a new sterile 15 mL centrifuge tube and washed with PBS, twice (500 g, 5 min), and finally the PBMCs were resuspended in 300 μL of PBS before use in the device.

The mesh-bound cells were fixed with 4% paraformaldehyde and washed with PBS. The fixed cells were infiltrated with 1% NP40 and blocked with 2% normal goat serum/3% BSA. Staining to identify CTCs was performed using well-established protocols using pan-Ck (Alexa Fluor488 anticytokeratin (CK, pan-reactive) antibody, Biolegend (628608)), CD45 (PE antihuman CD45 antibody, Biolegend (304008)), and DAPI (Sigma (D9542)). Secondary immunofluorescence labeled antibodies were used for the identification of PD-L1 positive cells: antihuman PD-L1 (Biolegend: 329708). Alexa Fluor 647 antiErbB2/HER2 antibody [EPR19547-12] (ab225510) was used for the identification of HER2 positive CTCs. After staining, the plate was washed with PBS and stored at 4 °C until microscopic imaging.

### Tissue Embedding Sectioning

The fresh patient biopsies were fixed in 4% formalin/paraformaldehyde and dehydrated in an ethanol series. After clearing in xylene, samples were infiltrated with paraffin wax. The wax block was cooled at −20 °C and sliced on a microtome in 4 μm sections. For immunostaining and FISH, sections were mounted, deparaffinized, and rehydrated.

### Immunohistochemistry (IHC)

Antigens were recovered in citric acid buffer (pH 6), and endogenous peroxidase activity as well as unspecific binding was blocked by transferring the sections into 3% BSA buffer. After 30 min, BSA was removed and sections were incubated with primary antibody (1:200, Alexa Fluor 647 antiErbB2/HER2 antibody [EP1045Y] (ab281578, Abcam, U.K.)). After overnight incubation at 4 °C, sections were washed with PBS and the secondary antibody (1:1000, Goat-Anti-Rabbit-HRP labeled, Servicebio, China) was applied at room temperature for 50 min. Again, sections were washed with PBS and stained with DAB staining kit (G1211, Servicebio, China) according to the manufacturer instructions. Nuclei in the sections were counterstained with hematoxylin. After being dehydrated and mounted, the stained tissue sections were visualized using a light microscope at x20 magnification.

### FISH Protocol

The FDA approved PathVysion HER2 DNA probe kit (Abbott Molecular, IL, USA) was used according to the manufacturer protocols. Briefly, DNA on slides was denatured at 72 ± 1 °C for 5 min and then washed and desiccated. After that, 10 μL of probe mixture was applied in a prewarmed humidified hybridization chamber at 37 ± 1 °C for 14–18 h. After hybridization, the sections were washed with SSC at 72 ± 1 °C and desiccated in the dark. 10 μL of DAPI was applied to counterstain the sections area of the slide. Sections were observed under a fluorescence microscope. The analyses of IHC and FISH were obtained from clinicians according to clinical guidelines.

### Statistical Analysis

Results were analyzed using Student’s two tailed *t*-test and ANOVA with equal or unequal variance in Minitab 19 (Minitab Inc., State College, PA, USA). Differences with *p*-values <0.05 were considered significant, and post-hoc Tukey’s tests were performed after significant ANOVA differences. The logistic regression and ROC curves were obtained using the scikit-learn Python package (Python 3, on Jupyter Notebook).

### Multiphysics Simulation

We used COMSOL Multiphysics (version 5.5) to conduct our simulations. To evaluate the effect of the flow rate on capture efficiency (Figure 3a), we used the “creeping flow” and “transport diluted species” modules with mesh boundary conditions (General Form Boundary PDE) to include a local Langmuir adsorption model as explained elsewhere.^54^ To evaluate time-dependent discrete events (Figure 3b), we selected the “creeping flow” and “particle tracing for fluid flow” modules. A “pass through” boundary condition was set on the outer perimeters of the channel, and a “stick” condition for the mesh, so particle–wall interactions could easily be determined visually. The study on the flow velocity (Figure 3c) was done using the creeping flow module. Each study was repeated at least four times.

## Supporting Information Available

Supporting text on the flow rate and fluid velocity dependence in microfluidic channels, 12 supplementary figures supporting the results, and two tables on the demographic details of the clinical study population (PDF)

## Notes

The study was conducted in accordance with the Declaration of Helsinki (as revised in 2013). The study was approved by the Ethics Committee of Fudan University Shanghai Cancer Center and Changzheng Hospital with individual consent.
