# Correction to “Flow Rate-Independent Multiscale Liquid Biopsy for Precision Oncology”

**DOI:** 10.1021/acssensors.3c01053

**Published:** 2023-06-07

**Authors:** Jie Wang, Robert Dallmann, Renquan Lu, Jing Yan, Jérôme Charmet

The authors regret that some errors were made on page 1202:

The sentence

*This notation emphasizes the flow rate limitation. Indeed, the equation suggests that flow rate cannot be increased without decreasing the Peclet number (and hence the capture efficiency).*

should be replaced by

*This notation emphasizes the flow rate limitation. Indeed, the equation suggests that flow rate cannot be increased without***increasing***the Peclet number.*

And the sentence

*Here we use meshes with microscale pore size and macroscale diameter mounted in a channel of matching size. In this configuration, the velocity can be kept low (to maintain Pe ≫ 1 and hence optimal capture) for any flow rate.*

should be replaced by

*Here we use meshes with microscale pore size and macroscale diameter mounted in a channel of matching size. In this configuration, the velocity can be kept low (to maintain Pe***≪***1 and hence optimal capture) for any flow rate.*

The authors would like to apologize for any inconvenience caused.

