# Supplementary material for: Flow Rate-Independent Multiscale Liquid Biopsy for Precision Oncology
Source: ACS Sens. 2023 Feb 20;8(3):1200–10. doi: 10.1021/acssensors.2c02577 (PMC10043932; doi:10.1021/acssensors.2c02577)
Supplement: Supplementary file 1 — se2c02577_si_001.pdf [file se2c02577_si_001.pdf]

Supplementary Materials for

Flow Rate Independent Multiscale Liquid Biopsy for Precision Oncology

Jie Wang[1], Robert Dallmann[2], Renquan Lu[3], Jing Yan*[4], and Jérôme Charmet*[2,5,6]

[1] Institute for Advanced Materials, School of Material Science and Engineering, Jiangsu University, Zhenjiang 212013, China,
[2] Division of Biomedical Sciences, Warwick Medical School, University of Warwick, Coventry CV4 7AL, United Kingdom,
[3] Department of Clinical Laboratory, Fudan University Shanghai Cancer Center, Shanghai, 200032, China,
[4] Holosensor Medical Technology Ltd., Suzhou, 215000, China,
[5] WMG University of Warwick, Coventry CV4 7AL, United Kingdom,
[6] School of Engineering – HE-Arc Ingénierie, HES-SO University of Applied Sciences Western Switzerland, 2000 Neuchâtel, Switzerland.

* corresponding authors: yj@holosmed.com (+86 400 1816 488) and jerome.charmet@he-arc.ch (+41 32 930 2629)

This PDF file includes:

Supplementary Text
Figs. S1 to S12
Tables S1 to S2

Supplementary Text

Flow rate and fluid velocity dependence in microfluidic channels

In affinity-capture microfluidic devices, the effective cross-section A is constrained by the micron length scale, for optimized capture efficiency, in one dimension and by the microfabrication processes (e.g., aspect ratio) or practical considerations (structural stability, etc.) in the other. In other words, the flow rate Q and the fluid velocity v are coupled through the square of the length scale, as given by $v \propto (Q/L)^2$. Therefore, any attempt to reduce the fluid velocity results in a lower interaction probability. Indeed, given a flow rate Q, the fluid velocity v can be decreased through an increase of the channel in cross-section A (as given by $v=Q/A$). However, this increases the length scale L (since $A \propto L^2$), which in turn decreases the interaction probability between cells and surface.

Fig. S1.

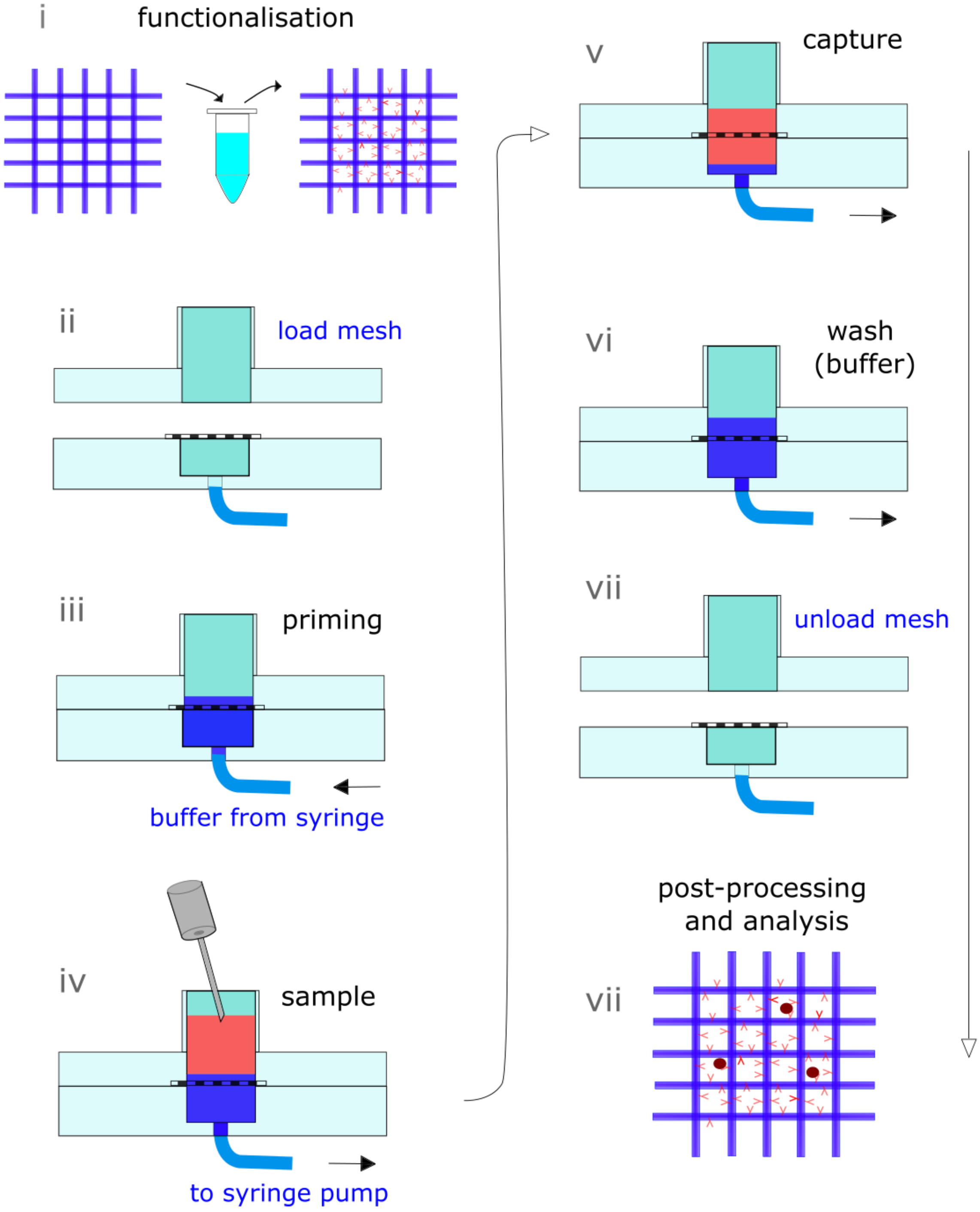

Figure S1. Schematic of process steps from pre-processing (functionalisation) to post-processing (e.g., fluorescence microscopy).

Fig. S2.

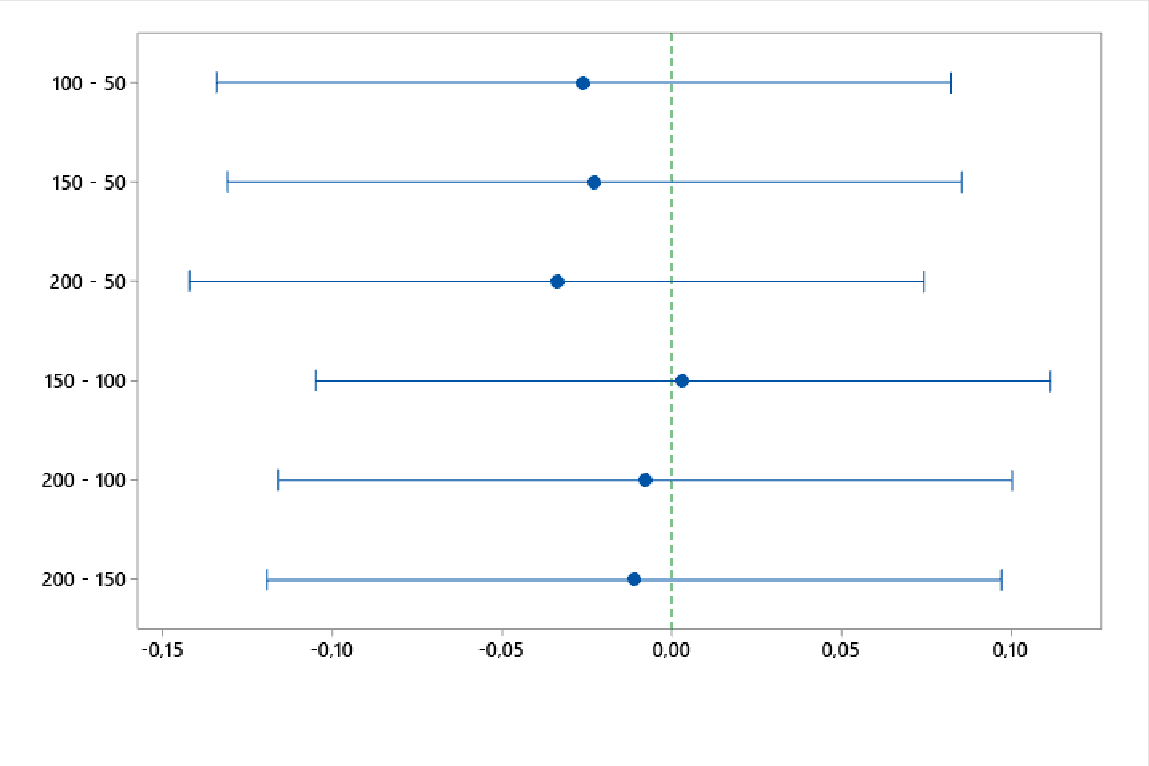

Figure S2. Graphs showing the post-hoc Tukey's test on flow rate independent data (Fig 2a). Each set (flow rate in μL min^{-1}) includes zero, which means that there is no statistical difference between any of the sets.

Fig. S3.

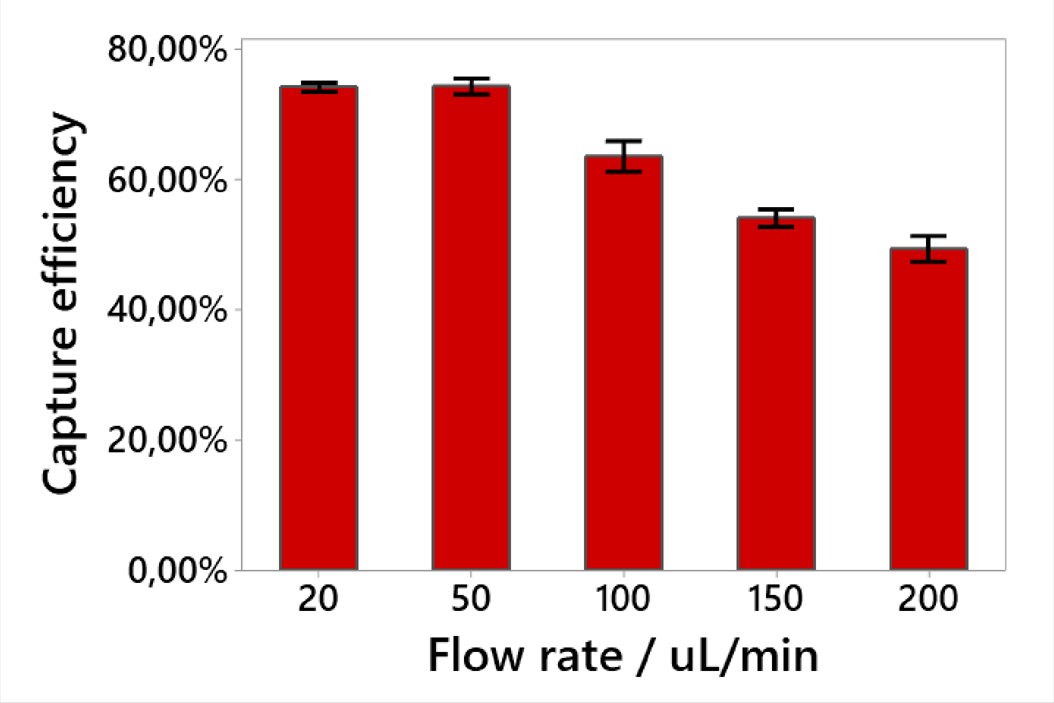

Figure S3. Graph showing the flow rate dependence of the device with a fixed diameter (6 mm in this case). An extra flow rate (20 µL min^{-1}) was added here to show the drop-off point at 50 µL min^{-1}, compared to Fig. 2.

Fig. S4.

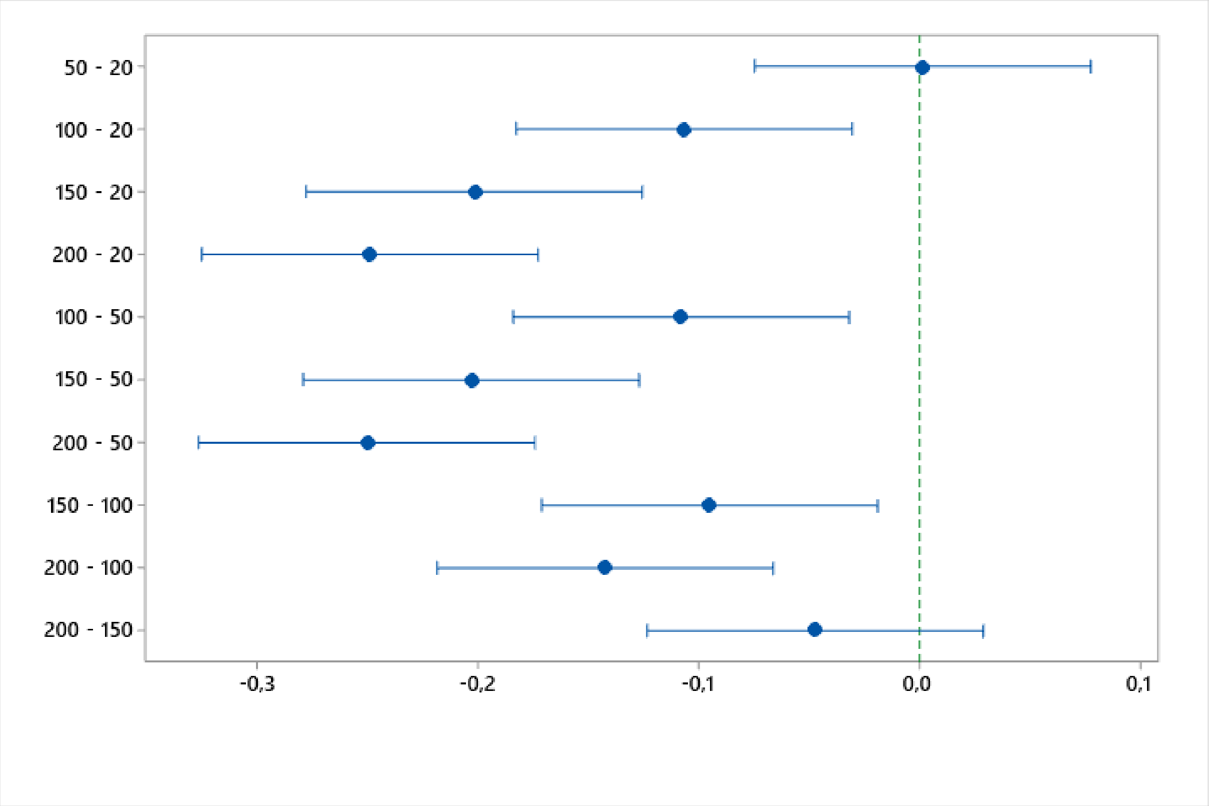

Figure S4. Graphs showing the post-hoc Tukey's test on flow rate dependent data (Fig S3 above). Two sets (20 – 50 µL min^{-1} and 200 – 150 µL min^{-1}) include zero, which means that there is no statistical difference between any of the sets. The rest of the sets are significantly different, confirming flow rate dependence on the capture efficiency.

Fig. S5.

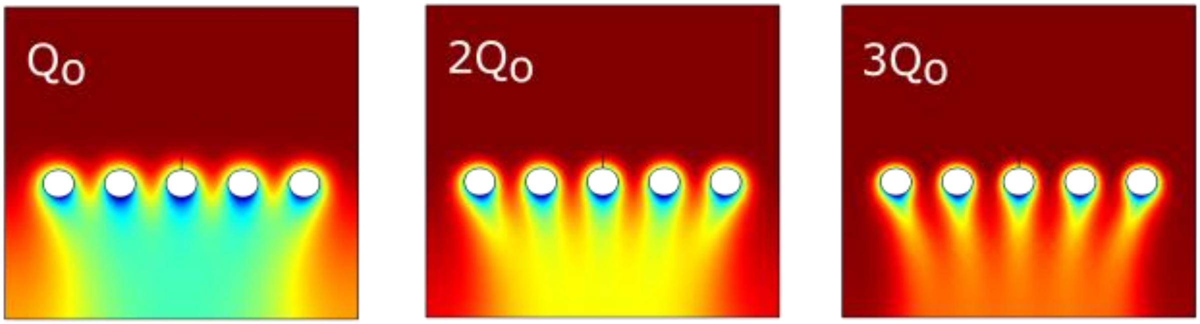

Figure S5. Additional simulation results. The effect of the flow rate on the capture efficiency can be observed on the 2D simulation that show the reaction of diluted species on the mesh (cross section representation). A diluted solution of particles (arbitrary concentration $C_{0)}$ is represented in red and introduced at the top of the channel at arbitrary flow rates Q_0, $2Q_0$ and $3Q_0$. The background solution is represented in blue ($C_0 = 0$). The blue traces therefore represent the solution depleted from the particles captured on the mesh. The simulations show that the capture efficiency decreases with increasing flow rate (i.e., less material is captured by the mesh) for a given channel diameter, which confirms our experimental results (Fig 2b).

Fig. S6.

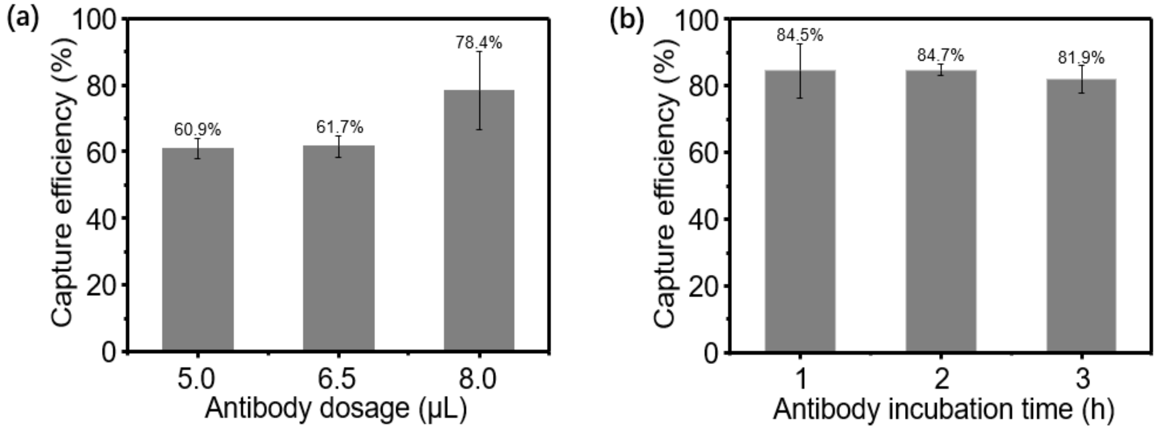

Figure S6. Antibody optimisation against capture efficiency (MCF-7 cells). The capture efficiency increases significantly for an 8 μL antibody dose compared 6.5 μL and 5 μL (a). This value was chosen for further analyses. Different incubation times between the polymer and anti-EpCAM antibodies were tested (b). No significant differences were observed for incubation times between 1h to 3h. The capture efficiency increases significantly for an 8 μL antibody dose compared 6.5 μL and 5 μL (a). This value was chosen for further analyses. Different incubation times between the polymer and anti-EpCAM antibodies were tested (b). No significant differences were observed for incubation times between 1h to 3h.

Fig. S7.

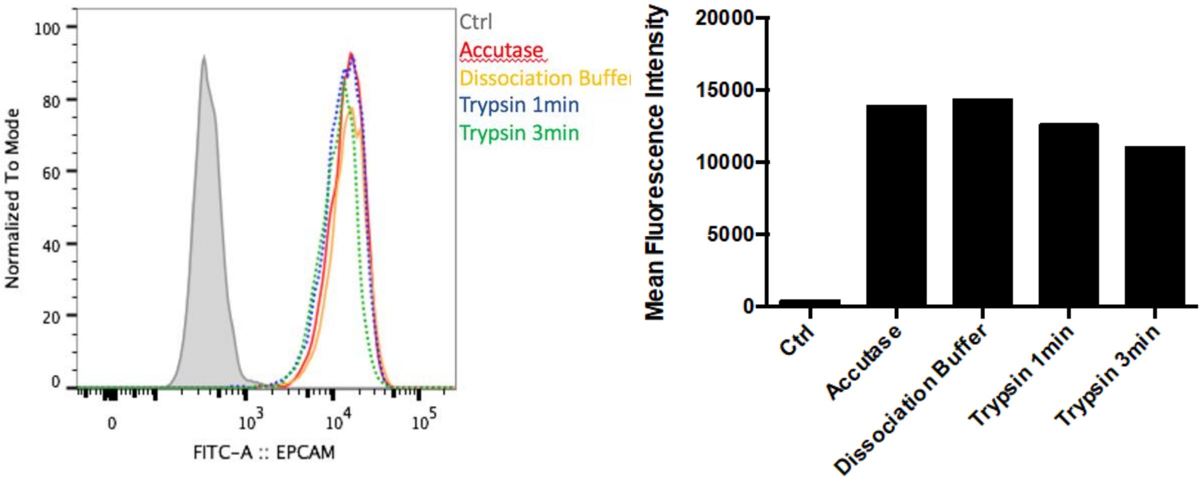

Figure S7. Control and optimisation of harvesting parameters and antibody selection. MCF-7 cells were stained with FITC anti-EPCAM (Clone: 9C4, Biolegend) and their EPCAM expression level was evaluated using flow cytometry. Other antibodies were tested but did not produce better results – not shown. Different harvesting reagents and parameters were tested to evaluate their effect on EpCAM expression. The effects of cell dissociation buffer in PBS, Accutase, 0.25% Trypsin (1 and 3 minutes) under standard conditions were characterised. Our results show that cell dissociation buffer in PBS or Accutase provide a gentler process, maintaining EpCAM integrity.

Fig. S8.

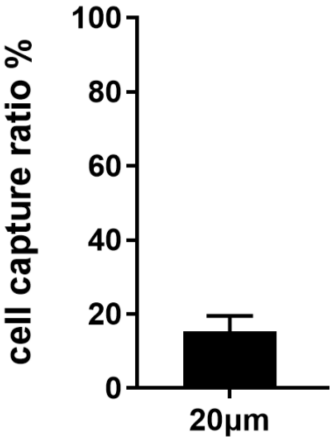

Figure S8. Traut reagent was used as a functionalisation strategy to tether the anti-EpCAM antibodies onto the gold coated mesh. However, the capture efficiency, presented here for a 20 x 20 µm pore size in a 6 mm diameter mesh at 50 µL min^{-1}, shows less than 20% capture efficiency, compared to over 60% in the same conditions with our HA-SH nanobranched polymer (Fig. 4C).

Fig. S9.

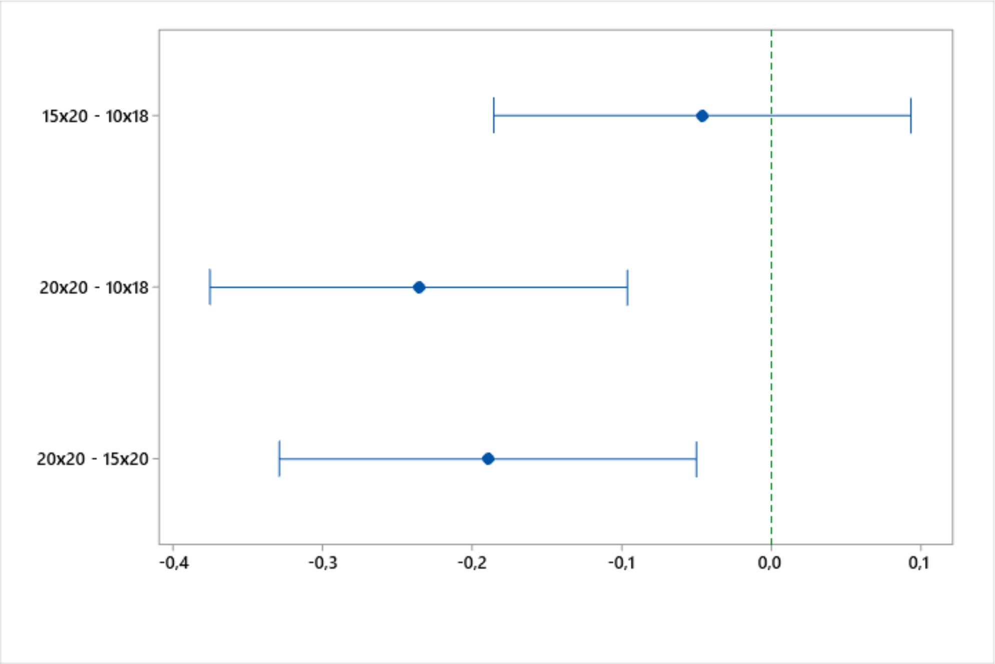

Figure S9. Graphs showing the post-hoc Tukey's test on the capture efficiency as a function of pore sizes. Figure 4c shows that the capture efficiency increases with decreasing pore sizes, however the graph above shows that the set (10x18 – 15x20 µm) includes zero, which means that there is no statistical difference between the two sets.

Fig. S10.

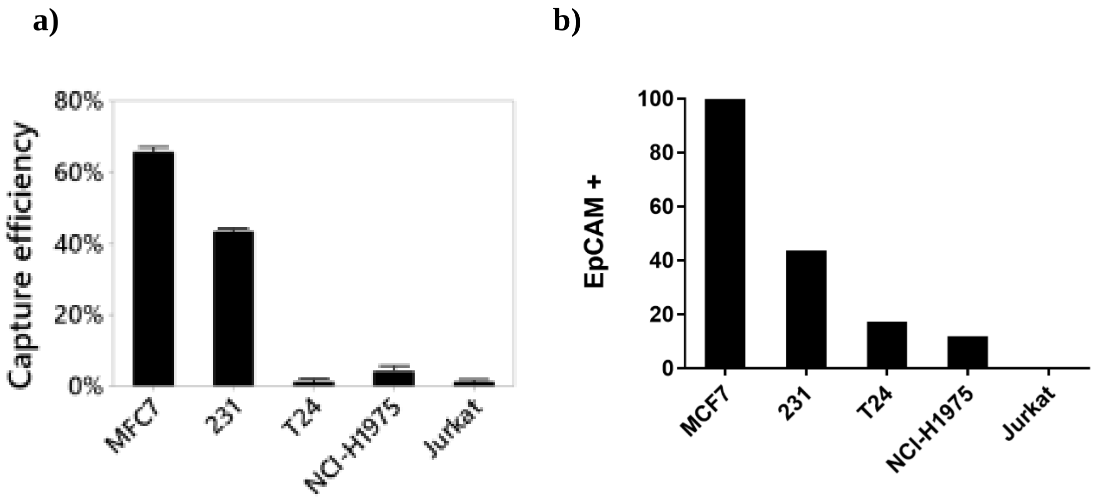

Figure S10. We measured the capture efficiency of MCF-7 cells with MDA-MB-231, T24, NCl-H1975 and Jurkat cells (a). The capture efficiencies were in agreement with the EpCAM expression level of each cell type, as measured using flow cytometry. The data of each cell type is relative to the EpCAM expression level of MCF7, which is set to 100%. The results are in line with data from the gene expression atlas (Gene ID ENSG00000119888) (b).

Fig. S11.

a) **b)**

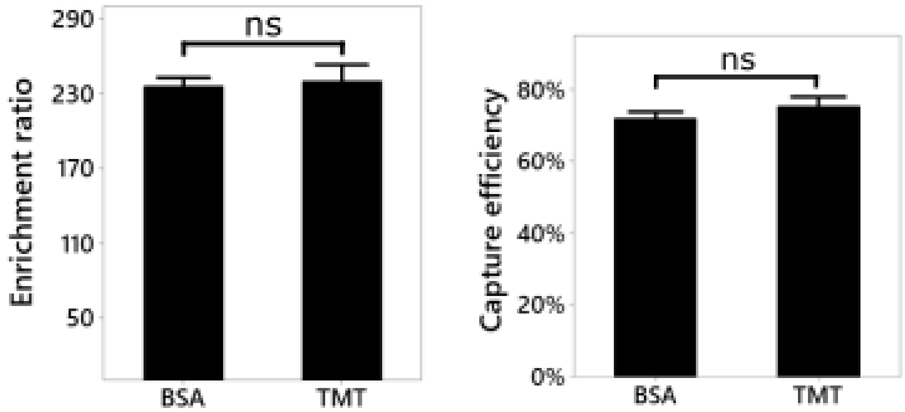

Figure S11. Effect of trimethoxylsilane (50%) blocking molecules. Compared to BSA blocking molecule, using the approach reported in the manuscript, we did not observe any significant improvement in enrichment (a) or changes in capture efficiency (b) for meshes with 15 x 20 μm pore size.

Fig. S12.

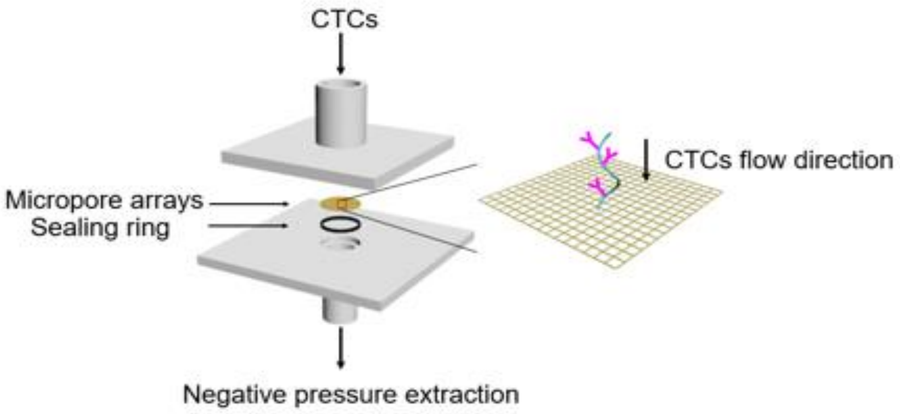

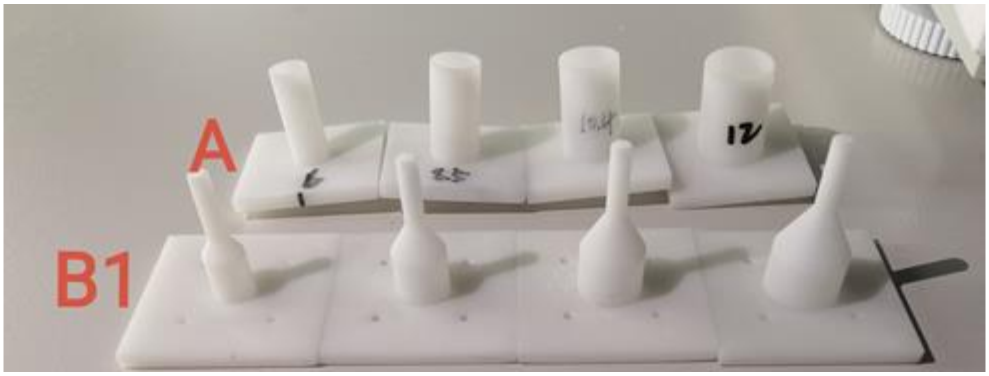

Figure S12. Device architecture and mesh holder. A schematic representing the assembly of the mesh in the holder (top) and different mesh holders used to evaluate the flow rate independence are shown (bottom).

Table S1.

Sample	Gender	Age	Date	Diagnosis	Detection type	Number		Rate
						CTC	CTC PD-L1	
1	F	61	2019.07.29	NSCLC	CTC+ PD-L1	5	2	40.0%
2	M	53	2019.08.01	NSCLC	CTC+ PD-L1	3	1	33.3%
3	F	60	2019.08.01	NSCLC	CTC+ PD-L1	2	0	0.0%
4	M	51	2019.08.04	NSCLC	CTC+ PD-L1	5	0	0.0%
5	M	84	2019.08.25	NSCLC	CTC+ PD-L1	7	0	0.0%
6	F	49	2019.09.03	NSCLC	CTC+ PD-L1	2	0	0.0%
7	M	46	2019.09.05	NSCLC	CTC+ PD-L1	6	2	33.3%
8	F	58	2019.11.24	NSCLC	CTC+ PD-L1	6	0	0.0%
9	M		2019.11.25	NSCLC	CTC+ PD-L1	4	0	0.0%
10	M		2019.11.28	NSCLC	CTC+ PD-L1	4	0	0.0%
11	F	47	2019.12.09	NSCLC	CTC+ PD-L1	2	1	50.0%
12	M	68	2019.12.26	NSCLC	CTC+ PD-L1	4	1	25.0%
13	M		2019.12.27	NSCLC	CTC+ PD-L1	2	1	50.0%
14	M		2019.12.28	NSCLC	CTC+ PD-L1	2	1	50.0%
15	F		2019.12.28	NSCLC	CTC+ PD-L1	9	1	11.1%
16	F		2020.01.10	NSCLC	CTC+ PD-L1	3	1	33.3%
17	M		2020.01.10	NSCLC	CTC+ PD-L1	2	0	0.0%
18	M		2020.01.10	NSCLC	CTC+ PD-L1	3	0	0.0%
19	M		2020.03.16	NSCLC	CTC+ PD-L1	6	0	0.0%
20			2020.03.23	NSCLC	CTC+ PD-L1	4	0	0.0%
21	M	56	2020.04.17	colorectal cancer	CTC+ PD-L1	3	0	0.0%
22	F	62	2020.04.17	colorectal cancer	CTC+ PD-L1	3	2	66.7%
23	F	64	2020.05.09	colorectal cancer	CTC+ PD-L1	4	0	0.0%
24	F		2020.05.26	NSCLC	CTC+ PD-L1	5	4	80.0%
25	M	57	2020.06.05	SCLC	CTC+ PD-L1	3	2	66.7%
26			2020.06.05	liver cancer	CTC+ PD-L1	6	0	0.0%
27	M	51	2020.06.14	Pulmonary nodules	CTC+ PD-L1	3	0	0.0%
28			2020.06.05	liver cancer	CTC+ PD-L1	2	0	0.00%
29			2020.06.05	liver cancer	CTC+ PD-L1	6	0	0.00%
30			2020.06.30	liver cancer	CTC+ PD-L1	2	0	0.00%
31	F	76	2022.01.14	liver cancer	CTC+ PD-L1	1	0	0.00%
32	M	36	2022.02.15	liver cancer	CTC+ PD-L1	1	0	0.00%
33	F	74	2022.02.10	pancreatic carcinoma	CTC+ PD-L1	0	0	0.00%

Table S1 (ctd).

Sample	Gender	Age	Date	Diagnosis	Detection type	CTC number
1	M		2019.08.23	NSCLC	CTC	6
2	M		2019.08.23	NSCLC	CTC	4
3	M		2019.08.27	NSCLC	CTC	1
4	M		2019.08.27	NSCLC	CTC	3
5	M		2019.08.27	NSCLC	CTC	2
6	M		2019.08.27	NSCLC	CTC	1
7	M		2019.08.27	NSCLC	CTC	5
8	M		2019.08.30	NSCLC	CTC	4
9	F		2019.08.30	NSCLC	CTC	4
10	M		2019.08.30	NSCLC	CTC	2
11	M		2019.08.30	NSCLC	CTC	4
12	F		2019.09.13	NSCLC	CTC	5
13	M		2019.10.26	NSCLC	CTC	2
14	M	42	2019.12.24	nasopharynx cancer	CTC	14
15	F		2020.03.14	cancer of biliary duct	CTC	3
16			2020.06.30	liver cancer	CTC	2
17	F	40	2019.11.21	breast cancer	CTC	4
18	F	51	2020.01.02	breast cancer	CTC	5
19	F	68	2020.03.12	breast cancer	CTC	2
20	F	66	2020.03.12	breast cancer	CTC	2

Number	Sampling time	Age	Gender	CTC
1	2021.12.04	29	Male	0
2	2021.12.04	28	Male	0
3	2021.12.04	31	Female	0
4	2021.11.25	24	Male	0
5	2021.11.25	29	Male	0
6	2021.11.25	35	Male	0
7	2021.11.26	24	Female	0
8	2021.11.26	28	Female	0
9	2021.11.26	29	Male	0
10	2021.12.28	31	Female	0
11	2021.12.28	31	Male	0
12	2021.12.28	30	Male	0
13	2021.12.28	33	Male	0
14	2021.12.28	32	Male	0
15	2021.12.19	28	Male	0
16	2021.12.19	26	Male	0
17	2021.12.19	29	Female	0
18	2021.12.19	24	Female	0
19	2021.12.20	27	Male	0
20	2021.12.20	27	Male	0

Table S1 : Details of patients enrolled in the first study and raw results for CTCs counts only (top) and healthy volunteers (bottom).

Table S2.

Time	NO.	Age	Diagnosis				Tissue grade	T	N	M	TNM	IHC HER2	FISH HER-2	ER	PR	Size	CTC	HER-2 CTC
2020.09.24	**1**	74	Breast cancer	Malignant	Invasive	Ductal carcinoma	3	2	1	0	ⅡB	2+	(-)	80%，+	60%，+	1.3*1.3*1cm	2	0
2020.11.06	**2**	41	Breast cancer	Malignant	Invasive	Ductal carcinoma	2	0	0	0	0	2+	(-)	95%，+	60%，+	/	1	0
2020.11.06	**3**	77	Breast cancer	Malignant	Invasive	Ductal carcinoma	3	2	0	0	ⅡA	2+	(-)	80%，+	80%，+	2*1.8*1.5cm	1	0
2020.11.13	**4**	48	Breast cancer	Malignant	Invasive	Ductal carcinoma	3	2	0	0	ⅡA	3+	(+)	-	-	4*3.5*2cm	5	2
2020.11.13	**5**	60	Breast cancer	Malignant	Invasive	Ductal carcinoma	2	3	1	0	ⅢA	2+	(+)	-	-	5×4×2cm	11	6
2020.11.13	**6**	71	Breast cancer	Malignant	Invasive	Apocrine sweat gland carcinoma	2	1	0	0	Ⅰ	2+	(-)	-	-	1.2*0.9*0.8cm	8	6
2020.11.17	**7**	59	Breast cancer	Malignant	/	/	/	/	/	/	/	2+	(-)	90%，+	80%，+	/	5	0
2020.12.02	**8**	33	Breast cancer	Malignant	Invasive	Ductal carcinoma	2	/	/	/	/	2+	(-)	80%，+	90%，+	2.5×1.5×1.5cm	10	6
2020.12.02	**9**	63	Breast cancer	Malignant	Invasive	Ductal carcinoma	2	1	0	0	Ⅰ	2+	(+)	80%，+	1%	2×1×1cm	8	5
2020.12.03	**10**	41	Breast cancer	Malignant	Invasive	Ductal carcinoma	2	3	0	0	ⅡB	2+	(-)	80%，+	80%，+	6×3×2cm	5	0
2020.12.03	**11**	42	Breast cancer	Malignant	Invasive	Ductal carcinoma	2	/	/	/	/	2+	(-)	70%，+	70%，+	1.6×1.1×1.3cm	5	1

Time	NO.	Age	Diagnosis				Tissue grade	T	N	M	TNM	IHC HER2	FISH HER-2	ER	PR	Size	CTC	HER-2 CTC
2020.12.07	**12**	56	Breast cancer	Malignant	Invasive	Ductal carcinoma	3	/	/	/	/	2+	(-)	-	-	2×1.5×1 cm	2	1
2020.12.08	**13**	31	Breast cancer	Malignant	Invasive	Ductal carcinoma	3	/	/	/	/	2+	(+)	90%，+	80%，+	/	7	1
2020.12.09	**14**	27	Breast cancer	Malignant	/	/	/	2	2	1	Ⅳ	2+	(+)	-	-	/	0	0
2020.12.10	**15**	49	Breast cancer	Malignant	/	/	/	/	/	/	/	3+	(+)	70%，+	2%，	25×25×15 mm	3	0
2020.12.11	**16**	36	Breast cancer	Malignant	Invasive	Ductal carcinoma	3	1	1	0	ⅡA	2+	(-)	-	-	1.1×1×0.9cm	1	1
2020.12.11	**17**	48	Breast cancer	Malignant	Invasive	Ductal carcinoma	2	1	0	0	Ⅰ	2+	(-)	80%，+	80%，+	1.5×1×1 cm	5	0
2020.12.14	**18**	41	Breast cancer	Malignant	Invasive	Ductal carcinoma	2--3	2	1	0	ⅡB	2+	(+)	80%，+	80%，+	2.5×1.5×1.3 cm	5	0
2020.12.14	**19**	68	Breast cancer	Malignant	Invasive	Ductal carcinoma	3	/	/	/	/	2+	(+)	-	-	1.4×1.2×1cm	5	1
2020.12.15	**20**	56	Breast cancer	Malignant	Invasive	Ductal carcinoma	2	1	0	0	Ⅰ	2+	(-)	60%，+	1%，+	2×1.2×1.1cm	7	1
2020.12.15	**21**	65	Breast cancer	Malignant	Invasive	Ductal carcinoma	3	2	1	0	ⅡB	2+	(+)	90%，+	2%	2×2×2 cm	2	1
2020.12.16	**22**	36	Breast cancer	Malignant	Invasive	/	/	/	/	/	/	1+	(-)	10%	-	/	7	0
2020.12.21	**23**	47	Breast cancer	Malignant	Invasive	Ductal carcinoma	2	x	0	0		2+	(-)	80%，+	80%，+	/	0	0

Time	NO.	Age	Diagnosis				Tissue grade	T	N	M	TNM	IHC HER2	FISH HER-2	ER	PR	Size	CTC	HER-2 CTC
2020.12.22	**24**	49	Breast cancer	Malignant	Invasive	Ductal carcinoma	3	/	/	/	/	3+	(+)	-	-	2*2*1cm	7	1
2020.12.24	**25**	50	Breast cancer	Malignant	Invasive	/	2	/	/	/	/	2+	(-)	90%，+	90%，+	2×2×1cm	3	0
2020.12.28	**26**	43	Breast cancer	Malignant	Invasive	Lobular carcinoma	2	1	1	0	ⅡA	1+	(-)	80%，+	80%，+	2.5×2×1.5cm	1	1

Table S2 : Details of breast cancer patients tested for HER2 status using clinical standard procedures (IHC and FISH) performed on solid tissue biopsies.
